# (2,9-Dimethyl-1,10-phenanthroline-κ^2^ *N*,*N*′)(4-hydroxy­benzoato-κ^2^ *O*,*O*′)(nitrato-κ*O*)copper(II)

**DOI:** 10.1107/S1600536808034788

**Published:** 2008-10-31

**Authors:** Cui-Ping Zhai, Feng-Mei Yan, Pei-Zheng Zhao

**Affiliations:** a College of Chemistry and Chemical Engineering, Henan University, Kaifeng 475001, People’s Republic of China; b Department of Chemistry and Chemical Engineering, Huanghuai University, Zhumadian 463000, People’s Republic of China; c College of Chemistry and Environmental Science, Henan Normal University, Xinxiang 453007, People’s Republic of China

## Abstract

In the title compound, [Cu(C_7_H_5_O_3_)(NO_3_)(C_14_H_12_N_2_)], the Cu^II^ ion is five-coordinated in a slightly distorted square-pyramidal geometry by one O atom of a nitrate anion, two O atoms of a 4-hydroxy­benzoate anion, and two N atoms from a bidentate 2,9-dimethyl-1,10-phenanthroline (dmphen) ligand. In the crystal structure, inversion-related mol­ecules are linked into dimers by O—H⋯O hydrogen bonds. The packing is further stabilized by π–π inter­actions involving the benzene rings of the dmphen and hydroxy­benzoate units, with centroid–centroid distances of 3.4930 (14) or 3.5727 (14) Å.

## Related literature

For related structures, see: Xuan *et al.* (2007 ▶); Zhao *et al.* (2007 ▶); Okabe *et al.* (2007 ▶). For general background, see: Selvakumar *et al.* (2006 ▶).
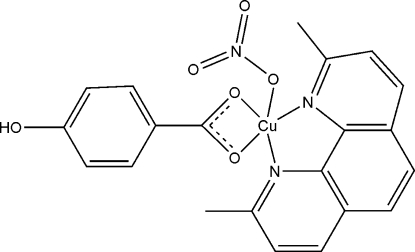


## Experimental

### 

#### Crystal data

[Cu(C_7_H_5_O_3_)(NO_3_)(C_14_H_12_N_2_)]
*M* *_r_* = 470.92
Triclinic, 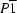

*a* = 9.594 (1) Å
*b* = 9.802 (1) Å
*c* = 12.347 (1) Å
α = 78.687 (14)°
β = 70.409 (13)°
γ = 63.740 (12)°
*V* = 979.4 (2) Å^3^
*Z* = 2
Mo *K*α radiation
μ = 1.16 mm^−1^
*T* = 291 (2) K
0.37 × 0.30 × 0.17 mm

#### Data collection

Bruker SMART CCD area-detector diffractometer
Absorption correction: multi-scan (*SADABS*; Bruker, 1997 ▶) *T*_min_ = 0.673, *T*_max_ = 0.830
7393 measured reflections
3613 independent reflections
3129 reflections with *I* > 2σ(*I*)
*R*_int_ = 0.016

#### Refinement

*R*[*F* ^2^ > 2σ(*F* ^2^)] = 0.031
*wR*(*F* ^2^) = 0.082
*S* = 1.02
3613 reflections
283 parameters
H-atom parameters constrained
Δρ_max_ = 0.29 e Å^−3^
Δρ_min_ = −0.28 e Å^−3^

### 

Data collection: *SMART* (Bruker, 1997 ▶); cell refinement: *SAINT* (Bruker, 1997 ▶); data reduction: *SAINT*; program(s) used to solve structure: *SHELXS97* (Sheldrick, 2008 ▶); program(s) used to refine structure: *SHELXL97* (Sheldrick, 2008 ▶); molecular graphics: *SHELXTL* (Sheldrick, 2008 ▶); software used to prepare material for publication: *SHELXTL*.

## Supplementary Material

Crystal structure: contains datablocks I, global. DOI: 10.1107/S1600536808034788/ci2692sup1.cif

Structure factors: contains datablocks I. DOI: 10.1107/S1600536808034788/ci2692Isup2.hkl

Additional supplementary materials:  crystallographic information; 3D view; checkCIF report

## Figures and Tables

**Table 1:** Selected bond lengths (Å)

Cu1—O1	2.004 (3)
Cu1—N1	2.007 (3)
Cu1—N2	2.008 (3)
Cu1—O2	2.026 (2)
Cu1—O4	2.292 (3)

**Table 2:** Hydrogen-bond geometry (Å, °)

*D*—H⋯*A*	*D*—H	H⋯*A*	*D*⋯*A*	*D*—H⋯*A*
O3—H3⋯O4^i^	0.82	1.97	2.767 (4)	164

Symmetry code: (i) 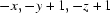.

**Fig. 1.**
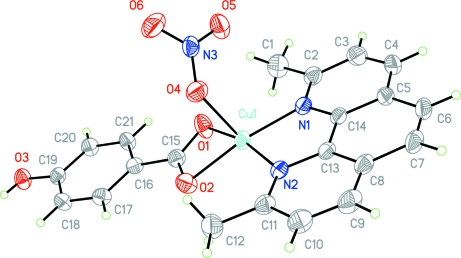
The molecular structure of the title complex, with atom labels and 30% probability displacement ellipsoids.

**Fig. 2.**
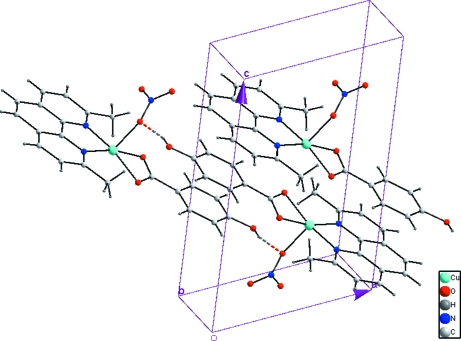
The hydrogen-bonding motifs, and π-π interactions between the aromatic rings of neighboring molecules in the crystal structure of the title compound. Dashed lines indicate the hydrogen bonds.

## supplementary crystallographic information

### Comment

The construction of novel Cu^II^ complexes are important for the development of new therapeutic drug design because of their antitumor activity (Selvakumar *et al.*, 2006). A number of Cu^II^ complexes have been synthesized and their crystal structures have been reported (Okabe *et al.*, 2007; Xuan *et al.*,2007; Zhao *et al.*, 2007). The title compound was recently obtained from the reaction of copper nitrate, sodium 4-hydroxybenzoate and dmphen in an ethanol-water mixture, and its crystal structure is reported here (Fig.1).

The Cu^II^ atom lies in a slightly distorted square-pyramidal coordination environment with one nitrate anion coordinated in the apical position. A bidentate 4-hydroxybenzoate anion binds to the Cu^II^ atom as a chelate through two oxygen atoms of the carboxylate group, together with two N atoms of the dmphen, constitute the base of the pyramid. The corresponding bond lengths are listed in Table 1.

In the crystal structure, inversion related molecules are linked into a dimer by O—H···O hydrogen bonds (Fig. 2). The packing is further stabilized by π-π interactions involving the benzene rings of the dmphen (C5-C8/C13/C14; centroid Cg1) and hydroxybenzoate (C16-C21; centroid Cg2) ligands (Fig. 2). The Cg1···Cg1^ii^ and Cg1···Cg2^iii^ distances are 3.4930 (14) Å and 3.5727 (14) Å, respectively [symmetry codes: (ii) 1 - *x*,-*y*,1 - *z*; (iii) 2-x, -y, -z]. This combination of hydrogen bonds and stacking interactions builds a three-dimensional network architecture.

### Experimental

4-Hydroxybenzoic acid (0.1389 g, 1 mmol) and NaOH (0.0380 g, 1 mmol) were dissolved in distilled water (10 ml) and 10 ml of Cu(NO_3_)_2_.3H_2_O (0.1220 g, 1 mmol) was added. This solution was added to a solution of 2,9-dimethyl-1,10-phenanthroline hemihydrate (C_14_H_12_N_2_^.^0.5H_2_O, 0.1088 g, 0.5 mmol) in ethanol (10 ml). The mixture was stirred at 323 K and then refluxed for 5 h, cooled to room temperature and filtered. Green single crystals of the title compound appeared over a period of two weeks by slow evaporation at room temperature.

### Refinement

Methyl and hydroxy H atoms were placed in calculated positions, with C-H = 0.96 Å and O-H = 0.82 %A, and refined with free torsion angles to fit the electron density; *U_iso_*(H) = 1.5*U_eq_*(carrier). Other H atoms were placed in calculated positions, with C-H = 0.93 %A, and refined using the riding-model approximation with *U_iso_*(H) = 1.2*U_eq_*(C).

### Crystal data

**Table:**

[Cu(C_7_H_5_O_3_)(NO_3_)(C_14_H_12_N_2_)]	*Z* = 2
*M**_r_* = 470.92	*F*(000) = 482
Triclinic, *P*1	*D*_x_ = 1.597 Mg m^−^^3^
Hall symbol: -P 1	Mo *K*α radiation, λ = 0.71073 Å
*a* = 9.594 (1) Å	Cell parameters from 3492 reflections
*b* = 9.802 (1) Å	θ = 2.5–26.8°
*c* = 12.347 (1) Å	µ = 1.16 mm^−^^1^
α = 78.687 (14)°	*T* = 291 K
β = 70.409 (13)°	Block, green
γ = 63.740 (12)°	0.37 × 0.30 × 0.17 mm
*V* = 979.4 (2) Å^3^	

### Data collection

**Table:**

Bruker SMART CCD area-detector diffractometer	3613 independent reflections
Radiation source: fine-focus sealed tube	3129 reflections with *I* > 2σ(*I*)
graphite	*R*_int_ = 0.016
φ and ω scans	θ_max_ = 25.5°, θ_min_ = 2.5°
Absorption correction: multi-scan (SADABS; Bruker, 1997)	*h* = −11→11
*T*_min_ = 0.673, *T*_max_ = 0.830	*k* = −11→11
7393 measured reflections	*l* = −14→14

### Refinement

**Table:**

Refinement on *F*^2^	Primary atom site location: structure-invariant direct methods
Least-squares matrix: full	Secondary atom site location: difference Fourier map
*R*[*F*^2^ > 2σ(*F*^2^)] = 0.031	Hydrogen site location: inferred from neighbouring sites
*wR*(*F*^2^) = 0.082	H-atom parameters constrained
*S* = 1.02	*w* = 1/[σ^2^(*F*_o_^2^) + (0.0426*P*)^2^ + 0.3425*P*] where *P* = (*F*_o_^2^ + 2*F*_c_^2^)/3
3613 reflections	(Δ/σ)_max_ = 0.001
283 parameters	Δρ_max_ = 0.29 e Å^−^^3^
0 restraints	Δρ_min_ = −0.28 e Å^−^^3^

### Special details

**Table:**

Geometry. All e.s.d.'s (except the e.s.d. in the dihedral angle between two l.s. planes)are estimated using the full covariance matrix. The cell e.s.d.'s are takeninto account individually in the estimation of e.s.d.'s in distances, anglesand torsion angles; correlations between e.s.d.'s in cell parameters are onlyused when they are defined by crystal symmetry. An approximate (isotropic)treatment of cell e.s.d.'s is used for estimating e.s.d.'s involving l.s. planes.
Refinement. Refinement of *F*^2^ against ALL reflections. The weighted *R*-factor *wR* andgoodness of fit *S* are based on *F*^2^, conventional *R*-factors *R* are basedon *F*, with *F* set to zero for negative *F*^2^. The threshold expression of*F*^2^ > σ(*F*^2^) is used only for calculating *R*-factors(gt) *etc*. and isnot relevant to the choice of reflections for refinement. *R*-factors basedon *F*^2^ are statistically about twice as large as those based on *F*, and *R*-factors based on ALL data will be even larger.

### Fractional atomic coordinates and isotropic or equivalent isotropic displacement parameters (Å^2^)

**Table:**

	*x*	*y*	*z*	*U*_iso_*/*U*_eq_	
Cu1	0.56944 (3)	0.10556 (3)	0.31286 (2)	0.04415 (11)	
O1	0.3621 (2)	0.0772 (2)	0.38002 (17)	0.0671 (5)	
O2	0.4119 (2)	0.2318 (2)	0.44919 (15)	0.0621 (5)	
O3	−0.33023 (19)	0.4060 (2)	0.72945 (16)	0.0582 (4)	
H3	−0.3541	0.4863	0.7561	0.087*	
O4	0.4677 (2)	0.3057 (2)	0.18943 (16)	0.0622 (5)	
O5	0.4474 (2)	0.1655 (2)	0.08484 (18)	0.0701 (5)	
O6	0.3103 (3)	0.4068 (3)	0.0818 (2)	0.1043 (9)	
N1	0.7212 (2)	−0.0741 (2)	0.21653 (16)	0.0421 (4)	
N2	0.7676 (2)	0.1464 (2)	0.28006 (16)	0.0422 (4)	
N3	0.4092 (2)	0.2916 (2)	0.11642 (18)	0.0504 (5)	
C1	0.5285 (3)	−0.1813 (3)	0.2297 (3)	0.0715 (8)	
H1A	0.5038	−0.2040	0.3114	0.107*	
H1B	0.5249	−0.2569	0.1929	0.107*	
H1C	0.4503	−0.0827	0.2133	0.107*	
C2	0.6932 (3)	−0.1819 (3)	0.1857 (2)	0.0510 (6)	
C3	0.8195 (4)	−0.2957 (3)	0.1132 (2)	0.0641 (7)	
H3A	0.7983	−0.3690	0.0920	0.077*	
C4	0.9713 (3)	−0.3004 (3)	0.0738 (2)	0.0625 (7)	
H4	1.0537	−0.3774	0.0270	0.075*	
C5	1.0041 (3)	−0.1884 (3)	0.10370 (19)	0.0496 (6)	
C6	1.1587 (3)	−0.1807 (3)	0.0638 (2)	0.0602 (7)	
H6	1.2452	−0.2542	0.0160	0.072*	
C7	1.1814 (3)	−0.0682 (3)	0.0943 (2)	0.0598 (7)	
H7	1.2828	−0.0648	0.0666	0.072*	
C8	1.0515 (3)	0.0449 (3)	0.1683 (2)	0.0498 (6)	
C9	1.0668 (3)	0.1644 (4)	0.2035 (2)	0.0643 (7)	
H9	1.1659	0.1724	0.1787	0.077*	
C10	0.9371 (3)	0.2677 (4)	0.2736 (3)	0.0665 (7)	
H10	0.9485	0.3463	0.2964	0.080*	
C11	0.7859 (3)	0.2593 (3)	0.3126 (2)	0.0522 (6)	
C12	0.6441 (3)	0.3759 (3)	0.3901 (3)	0.0737 (8)	
H12A	0.5606	0.4308	0.3523	0.111*	
H12B	0.6781	0.4456	0.4075	0.111*	
H12C	0.6030	0.3258	0.4602	0.111*	
C13	0.8981 (2)	0.0411 (3)	0.20915 (18)	0.0406 (5)	
C14	0.8733 (3)	−0.0774 (2)	0.17599 (18)	0.0410 (5)	
C15	0.3136 (3)	0.1778 (3)	0.4508 (2)	0.0473 (5)	
C16	0.1466 (3)	0.2347 (3)	0.52814 (18)	0.0417 (5)	
C17	0.0886 (3)	0.3593 (3)	0.59319 (19)	0.0470 (5)	
H17	0.1576	0.4037	0.5909	0.056*	
C18	−0.0697 (3)	0.4184 (3)	0.6611 (2)	0.0468 (5)	
H18	−0.1069	0.5020	0.7041	0.056*	
C19	−0.1735 (3)	0.3527 (3)	0.66513 (19)	0.0431 (5)	
C20	−0.1163 (3)	0.2265 (3)	0.6021 (2)	0.0496 (6)	
H20	−0.1845	0.1806	0.6060	0.060*	
C21	0.0425 (3)	0.1690 (3)	0.5334 (2)	0.0481 (5)	
H21	0.0798	0.0855	0.4903	0.058*	

### Atomic displacement parameters (Å^2^)

**Table:**

	*U*^11^	*U*^22^	*U*^33^	*U*^12^	*U*^13^	*U*^23^
Cu1	0.03013 (15)	0.04953 (18)	0.05171 (18)	−0.01577 (12)	−0.00692 (11)	−0.00997 (12)
O1	0.0446 (9)	0.0701 (12)	0.0827 (13)	−0.0273 (9)	0.0080 (9)	−0.0326 (10)
O2	0.0428 (9)	0.0921 (14)	0.0585 (10)	−0.0370 (9)	0.0016 (8)	−0.0242 (9)
O3	0.0380 (9)	0.0583 (11)	0.0737 (12)	−0.0203 (8)	−0.0022 (8)	−0.0164 (9)
O4	0.0612 (11)	0.0592 (11)	0.0719 (12)	−0.0204 (9)	−0.0303 (10)	−0.0054 (9)
O5	0.0574 (11)	0.0630 (12)	0.0845 (14)	−0.0193 (9)	−0.0084 (10)	−0.0260 (10)
O6	0.1100 (19)	0.0705 (14)	0.129 (2)	0.0055 (13)	−0.0824 (17)	−0.0155 (14)
N1	0.0362 (9)	0.0428 (10)	0.0467 (10)	−0.0140 (8)	−0.0142 (8)	−0.0023 (8)
N2	0.0334 (9)	0.0479 (10)	0.0472 (10)	−0.0164 (8)	−0.0136 (8)	−0.0037 (8)
N3	0.0319 (10)	0.0550 (13)	0.0567 (12)	−0.0123 (9)	−0.0060 (9)	−0.0118 (10)
C1	0.0650 (17)	0.0609 (17)	0.105 (2)	−0.0342 (14)	−0.0248 (16)	−0.0169 (16)
C2	0.0521 (14)	0.0424 (13)	0.0617 (15)	−0.0162 (11)	−0.0243 (12)	−0.0028 (11)
C3	0.0733 (19)	0.0473 (14)	0.0741 (18)	−0.0150 (13)	−0.0308 (15)	−0.0147 (13)
C4	0.0585 (16)	0.0519 (15)	0.0574 (16)	0.0012 (12)	−0.0186 (13)	−0.0148 (12)
C5	0.0440 (13)	0.0516 (14)	0.0397 (12)	−0.0069 (10)	−0.0137 (10)	−0.0015 (10)
C6	0.0371 (13)	0.0738 (18)	0.0433 (14)	−0.0045 (12)	−0.0047 (10)	−0.0027 (12)
C7	0.0319 (12)	0.0829 (19)	0.0499 (14)	−0.0182 (12)	−0.0061 (10)	0.0056 (13)
C8	0.0356 (12)	0.0675 (16)	0.0433 (13)	−0.0215 (11)	−0.0122 (10)	0.0063 (11)
C9	0.0444 (14)	0.091 (2)	0.0704 (18)	−0.0419 (14)	−0.0157 (13)	0.0026 (15)
C10	0.0567 (16)	0.0772 (19)	0.084 (2)	−0.0401 (15)	−0.0211 (15)	−0.0098 (16)
C11	0.0445 (13)	0.0579 (15)	0.0634 (15)	−0.0236 (11)	−0.0204 (11)	−0.0074 (12)
C12	0.0550 (16)	0.0690 (18)	0.106 (2)	−0.0223 (14)	−0.0214 (16)	−0.0340 (17)
C13	0.0326 (11)	0.0506 (13)	0.0363 (11)	−0.0159 (9)	−0.0120 (9)	0.0038 (9)
C14	0.0344 (11)	0.0448 (12)	0.0385 (11)	−0.0107 (9)	−0.0139 (9)	0.0021 (9)
C15	0.0407 (12)	0.0554 (14)	0.0438 (13)	−0.0203 (11)	−0.0092 (10)	−0.0012 (11)
C16	0.0388 (11)	0.0499 (13)	0.0374 (11)	−0.0214 (10)	−0.0092 (9)	0.0005 (10)
C17	0.0432 (12)	0.0598 (14)	0.0459 (13)	−0.0306 (11)	−0.0088 (10)	−0.0033 (11)
C18	0.0475 (13)	0.0489 (13)	0.0458 (13)	−0.0230 (11)	−0.0068 (10)	−0.0093 (10)
C19	0.0355 (11)	0.0477 (12)	0.0433 (12)	−0.0172 (10)	−0.0091 (9)	0.0003 (10)
C20	0.0418 (12)	0.0523 (14)	0.0610 (15)	−0.0259 (11)	−0.0104 (11)	−0.0073 (11)
C21	0.0455 (13)	0.0465 (13)	0.0517 (14)	−0.0198 (10)	−0.0073 (10)	−0.0105 (10)

### Geometric parameters (Å, °)

**Table:**

Cu1—O1	2.004 (3)	C5—C6	1.429 (4)
Cu1—N1	2.007 (3)	C6—C7	1.356 (4)
Cu1—N2	2.008 (3)	C6—H6	0.93
Cu1—O2	2.026 (2)	C7—C8	1.425 (4)
Cu1—O4	2.292 (3)	C7—H7	0.93
Cu1—C15	2.357 (3)	C8—C13	1.402 (4)
O1—C15	1.269 (3)	C8—C9	1.406 (4)
O2—C15	1.264 (3)	C9—C10	1.352 (4)
O3—C19	1.353 (3)	C9—H9	0.93
O3—H3	0.82	C10—C11	1.402 (4)
O4—N3	1.266 (3)	C10—H10	0.93
O5—N3	1.228 (3)	C11—C12	1.501 (4)
O6—N3	1.221 (3)	C12—H12A	0.96
N1—C2	1.346 (3)	C12—H12B	0.96
N1—C14	1.363 (3)	C12—H12C	0.96
N2—C11	1.345 (3)	C13—C14	1.438 (3)
N2—C13	1.370 (3)	C15—C16	1.478 (3)
C1—C2	1.486 (4)	C16—C21	1.387 (3)
C1—H1A	0.96	C16—C17	1.390 (3)
C1—H1B	0.96	C17—C18	1.380 (3)
C1—H1C	0.96	C17—H17	0.93
C2—C3	1.408 (4)	C18—C19	1.390 (3)
C3—C4	1.354 (4)	C18—H18	0.93
C3—H3A	0.93	C19—C20	1.388 (4)
C4—C5	1.409 (4)	C20—C21	1.386 (3)
C4—H4	0.93	C20—H20	0.93
C5—C14	1.406 (3)	C21—H21	0.93
			
O1—Cu1—N1	105.07 (10)	C6—C7—H7	119.7
O1—Cu1—N2	167.57 (8)	C8—C7—H7	119.7
N1—Cu1—N2	84.14 (10)	C13—C8—C9	116.6 (2)
O1—Cu1—O2	64.92 (9)	C13—C8—C7	119.8 (2)
N1—Cu1—O2	161.37 (8)	C9—C8—C7	123.6 (2)
N2—Cu1—O2	103.84 (9)	C10—C9—C8	119.9 (2)
O1—Cu1—O4	94.09 (10)	C10—C9—H9	120.1
N1—Cu1—O4	106.75 (12)	C8—C9—H9	120.1
N2—Cu1—O4	91.11 (10)	C9—C10—C11	121.6 (3)
O2—Cu1—O4	90.11 (12)	C9—C10—H10	119.2
O1—Cu1—C15	32.57 (9)	C11—C10—H10	119.2
N1—Cu1—C15	136.29 (9)	N2—C11—C10	119.8 (2)
N2—Cu1—C15	136.17 (10)	N2—C11—C12	119.8 (2)
O2—Cu1—C15	32.40 (8)	C10—C11—C12	120.3 (2)
O4—Cu1—C15	91.14 (10)	C11—C12—H12A	109.5
C15—O1—Cu1	89.23 (15)	C11—C12—H12B	109.5
C15—O2—Cu1	88.38 (16)	H12A—C12—H12B	109.5
C19—O3—H3	109.5	C11—C12—H12C	109.5
N3—O4—Cu1	121.09 (17)	H12A—C12—H12C	109.5
C2—N1—C14	118.7 (2)	H12B—C12—H12C	109.5
C2—N1—Cu1	130.40 (18)	N2—C13—C8	123.0 (2)
C14—N1—Cu1	110.83 (15)	N2—C13—C14	117.3 (2)
C11—N2—C13	119.1 (2)	C8—C13—C14	119.7 (2)
C11—N2—Cu1	130.43 (16)	N1—C14—C5	123.5 (2)
C13—N2—Cu1	110.44 (16)	N1—C14—C13	117.20 (19)
O6—N3—O5	121.6 (2)	C5—C14—C13	119.3 (2)
O6—N3—O4	117.7 (2)	O2—C15—O1	117.3 (2)
O5—N3—O4	120.7 (2)	O2—C15—C16	121.2 (2)
C2—C1—H1A	109.5	O1—C15—C16	121.4 (2)
C2—C1—H1B	109.5	O2—C15—Cu1	59.23 (14)
H1A—C1—H1B	109.5	O1—C15—Cu1	58.19 (13)
C2—C1—H1C	109.5	C16—C15—Cu1	174.09 (17)
H1A—C1—H1C	109.5	C21—C16—C17	118.8 (2)
H1B—C1—H1C	109.5	C21—C16—C15	120.6 (2)
N1—C2—C3	120.2 (2)	C17—C16—C15	120.5 (2)
N1—C2—C1	119.6 (2)	C18—C17—C16	121.1 (2)
C3—C2—C1	120.1 (2)	C18—C17—H17	119.5
C4—C3—C2	121.3 (3)	C16—C17—H17	119.5
C4—C3—H3A	119.4	C17—C18—C19	119.8 (2)
C2—C3—H3A	119.4	C17—C18—H18	120.1
C3—C4—C5	119.8 (2)	C19—C18—H18	120.1
C3—C4—H4	120.1	O3—C19—C20	117.7 (2)
C5—C4—H4	120.1	O3—C19—C18	122.6 (2)
C14—C5—C4	116.5 (2)	C20—C19—C18	119.7 (2)
C14—C5—C6	119.4 (2)	C21—C20—C19	120.0 (2)
C4—C5—C6	124.1 (2)	C21—C20—H20	120.0
C7—C6—C5	121.1 (2)	C19—C20—H20	120.0
C7—C6—H6	119.4	C20—C21—C16	120.7 (2)
C5—C6—H6	119.4	C20—C21—H21	119.7
C6—C7—C8	120.6 (2)	C16—C21—H21	119.7
			
N1—Cu1—O1—C15	165.73 (15)	C13—N2—C11—C10	0.2 (4)
N2—Cu1—O1—C15	28.8 (4)	Cu1—N2—C11—C10	−176.80 (19)
O2—Cu1—O1—C15	2.50 (14)	C13—N2—C11—C12	179.9 (2)
O4—Cu1—O1—C15	−85.71 (18)	Cu1—N2—C11—C12	3.0 (4)
O1—Cu1—O2—C15	−2.51 (14)	C9—C10—C11—N2	−0.1 (4)
N1—Cu1—O2—C15	−63.2 (3)	C9—C10—C11—C12	−179.9 (3)
N2—Cu1—O2—C15	−176.87 (14)	C11—N2—C13—C8	0.0 (3)
O4—Cu1—O2—C15	91.96 (17)	Cu1—N2—C13—C8	177.49 (17)
O1—Cu1—O4—N3	−57.54 (18)	C11—N2—C13—C14	−179.8 (2)
N1—Cu1—O4—N3	49.54 (19)	Cu1—N2—C13—C14	−2.2 (2)
N2—Cu1—O4—N3	133.76 (17)	C9—C8—C13—N2	−0.1 (3)
O2—Cu1—O4—N3	−122.40 (18)	C7—C8—C13—N2	−179.7 (2)
C15—Cu1—O4—N3	−90.02 (19)	C9—C8—C13—C14	179.6 (2)
O1—Cu1—N1—C2	9.1 (2)	C7—C8—C13—C14	0.0 (3)
N2—Cu1—N1—C2	−179.4 (2)	C2—N1—C14—C5	−0.4 (3)
O2—Cu1—N1—C2	64.0 (3)	Cu1—N1—C14—C5	−178.41 (17)
O4—Cu1—N1—C2	−90.0 (2)	C2—N1—C14—C13	178.98 (19)
C15—Cu1—N1—C2	20.2 (3)	Cu1—N1—C14—C13	1.0 (2)
O1—Cu1—N1—C14	−173.18 (14)	C4—C5—C14—N1	−0.2 (3)
N2—Cu1—N1—C14	−1.68 (14)	C6—C5—C14—N1	178.8 (2)
O2—Cu1—N1—C14	−118.3 (2)	C4—C5—C14—C13	−179.5 (2)
O4—Cu1—N1—C14	87.72 (16)	C6—C5—C14—C13	−0.5 (3)
C15—Cu1—N1—C14	−162.11 (14)	N2—C13—C14—N1	0.9 (3)
O1—Cu1—N2—C11	−42.2 (5)	C8—C13—C14—N1	−178.84 (19)
N1—Cu1—N2—C11	179.3 (2)	N2—C13—C14—C5	−179.72 (19)
O2—Cu1—N2—C11	−17.8 (2)	C8—C13—C14—C5	0.6 (3)
O4—Cu1—N2—C11	72.6 (2)	Cu1—O2—C15—O1	4.0 (2)
C15—Cu1—N2—C11	−20.2 (3)	Cu1—O2—C15—C16	−173.1 (2)
O1—Cu1—N2—C13	140.6 (3)	Cu1—O1—C15—O2	−4.1 (2)
N1—Cu1—N2—C13	2.12 (14)	Cu1—O1—C15—C16	173.1 (2)
O2—Cu1—N2—C13	165.01 (14)	O1—Cu1—C15—O2	175.8 (2)
O4—Cu1—N2—C13	−104.60 (17)	N1—Cu1—C15—O2	155.62 (14)
C15—Cu1—N2—C13	162.59 (14)	N2—Cu1—C15—O2	4.4 (2)
Cu1—O4—N3—O6	155.5 (2)	O4—Cu1—C15—O2	−88.41 (18)
Cu1—O4—N3—O5	−22.2 (3)	N1—Cu1—C15—O1	−20.2 (2)
C14—N1—C2—C3	0.2 (3)	N2—Cu1—C15—O1	−171.39 (14)
Cu1—N1—C2—C3	177.79 (18)	O2—Cu1—C15—O1	−175.8 (2)
C14—N1—C2—C1	179.5 (2)	O4—Cu1—C15—O1	95.81 (18)
Cu1—N1—C2—C1	−3.0 (3)	O2—C15—C16—C21	−176.7 (2)
N1—C2—C3—C4	0.5 (4)	O1—C15—C16—C21	6.3 (4)
C1—C2—C3—C4	−178.7 (3)	O2—C15—C16—C17	6.2 (3)
C2—C3—C4—C5	−1.1 (4)	O1—C15—C16—C17	−170.8 (2)
C3—C4—C5—C14	0.9 (4)	C21—C16—C17—C18	−0.6 (3)
C3—C4—C5—C6	−178.0 (2)	C15—C16—C17—C18	176.6 (2)
C14—C5—C6—C7	−0.1 (4)	C16—C17—C18—C19	0.1 (4)
C4—C5—C6—C7	178.8 (2)	C17—C18—C19—O3	−179.5 (2)
C5—C6—C7—C8	0.7 (4)	C17—C18—C19—C20	1.0 (3)
C6—C7—C8—C13	−0.6 (4)	O3—C19—C20—C21	178.9 (2)
C6—C7—C8—C9	179.8 (2)	C18—C19—C20—C21	−1.6 (4)
C13—C8—C9—C10	0.1 (4)	C19—C20—C21—C16	1.1 (4)
C7—C8—C9—C10	179.7 (3)	C17—C16—C21—C20	0.0 (4)
C8—C9—C10—C11	0.0 (4)	C15—C16—C21—C20	−177.2 (2)

### Hydrogen-bond geometry (Å, °)

**Table:**

*D*—H···*A*	*D*—H	H···*A*	*D*···*A*	*D*—H···*A*
O3—H3···O4^i^	0.82	1.97	2.767 (4)	164

Symmetry codes: (i) −*x*, −*y*+1, −*z*+1.

## References

[bb1] Bruker (1997). *SMART*, *SAINT* and *SADABS* Bruker AXS Inc., Madison, Wisconsin, USA.

[bb2] Okabe, N., Tsuji, A. & Yodoshi, M. (2007). *Acta Cryst.* E**63**, m1756–m1757.

[bb3] Selvakumar, B., Rajendiran, V., Maheswari, P. U., Stoeckli-Evans, H. & Palaniandavar, M. (2006). *J. Inorg. Biochem.* **100**, 316–330. 10.1016/j.jinorgbio.2005.11.018 16406550

[bb4] Sheldrick, G. M. (2008). *Acta Cryst.* A**64**, 112–122. 10.1107/S0108767307043930 18156677

[bb5] Xuan, X.-P., Zhao, P.-Z. & Zhang, S.-X. (2007). *Acta Cryst.* E**63**, m1817.

[bb6] Zhao, P.-Z., Yan, F.-M., Xuan, X.-P. & Tang, Q.-H. (2007). *Acta Cryst.* E**63**, m2523.

